# Enhanced Pollutant Removal and Antifouling in an Aerobic Ceramic Membrane Bioreactor with Bentonite for Pharmaceutical Wastewater Treatment

**DOI:** 10.3390/membranes14100205

**Published:** 2024-09-26

**Authors:** Salaheddine Elmoutez, Hafida Ayyoub, Mohamed Chaker Necibi, Azzedine Elmidaoui, Mohamed Taky

**Affiliations:** 1 International Water Research Institute IWRI, Mohammed VI Polytechnic University, Lot 660, Ben Guerir 43150, Morocco; chaker.necibi@um6p.ma (M.C.N.); azzeddine.elmidaoui@um6p.ma (A.E.); 2 Laboratory of Advanced Materials and Process Engineering, Faculty of Sciences, Ibn Tofail University, Kenitra BP 1246, Morocco; hafida.ayyoub@uit.ac.ma (H.A.); mohamed.taky@uit.ac.ma (M.T.)

**Keywords:** pharmaceutical wastewater, bentonite, membrane bioreactors, nitrogen removal, heavy metal, adsorption, fouling mitigation

## Abstract

This study examined the impact of adding bentonite clay (concentration of 1.5 to 10 g/L) to a pilot-scale aerobic ceramic membrane bioreactor (AeCMBR) for treating pharmaceutical wastewater (PhWW). The hydraulic retention time (HRT) was maintained at 24 h; the dissolved oxygen was between 2 mg/L (on) and 4 mg/L (off) throughout operation. Organic and nitrogen pollution removal rates and heavy metal (Cu, Ni, Pb, Zn) reduction rates were assessed. The chemical oxygen demand (COD) removal efficiency exceeded 82%. Adsorption improved ammonia (NH4+) removal to 78%; the addition of 5 g of bentonite resulted in a 38% improvement compared with the process without bentonite. The average nitrate concentration decreased from 169.69 mg/L to 43.72 mg/L. The average removal efficiencies for Cu, Ni, Pb and Zn were 86%, 68.52%, 46.90% and 56.76%, respectively. Bentonite at 5 g/L significantly reduced membrane fouling. The cost–benefit analysis enabled us to predict that the process will meet the multiple objectives of durability, treatment performance and economic viability. The combination of an AeCMBR and bentonite adsorption has proven to be a valuable solution for treating highly polluted wastewater.

## 1. Introduction

Industrial activities pose a major threat to the natural environment. The rapid expansion of the pharmaceutical industry in recent decades has led to the extensive use of pharmaceuticals and veterinary medicines in various sectors such as agriculture, poultry, livestock, fisheries and human health [1]. While pharmaceuticals have brought many benefits to society, they have also caused considerable damage to the environment due to their structural stability and resistance to biodegradation [2,3]. The production of pharmaceutical products generates a considerable quantity and diversity of waste, ranging from 200 to 30,000 kg per kilogram of active ingredient produced, exceeding the quantity of the finished products [4]. The relevant manufacturing process consumes a significant amount of water, resulting in the release of harmful pollutants into aqueous effluents [5]. Pharmaceutical wastewater (PhWW) contains relatively high levels of suspended solids (SSs), soluble organic matter and micropollutants, many of which are unresponsive to conventional treatment [6,7].

In the north-western region of Morocco, surface waters frequently exhibit elevated concentrations of organic and nitrogenous pollutants and heavy metals from the industrial sector, thus posing potential risks to both the environment and human health. The removal and immobilization of these contaminants are of paramount importance to ensure the safety and well-being of the ecosystem.

Given the economic factors involved, biological treatment processes are appropriate for highly polluted industrial wastewater [8]. Ammonia nitrogen is removed via aerobic nitrification and anoxic denitrification, involving autotrophic nitrifying bacteria and heterotrophic denitrifying bacteria [9]. However, conventional treatment processes are often unable to effectively remove the pollutants present in PhWW and reduce them to acceptable levels [8]. It is therefore essential to find an alternative treatment option able to reduce organic pollution, nitrogen, and heavy metal concentrations to acceptable levels that is able to deal with high initial concentrations, easy to use and offers the potential for regeneration and reuse. Various technologies have been developed for the removal of organo-nitrogen pollutants and heavy metals, including ion exchange [10], photoelectrocatalysis [11], chemical precipitation [12], anammox [13], air stripping [14], biological treatment processes [15], and adsorption methods [16]. Membrane bioreactors (MBRs) are gaining increasingly more attention given their high capacity to treat municipal and industrial wastewater [17].

MBRs offer clear advantages over other biological systems, not only because they are more effective in disinfection, but also because they have a limited footprint, limited sludge generation, better effluent quality and longer sludge retention times (SRTs) (independent of hydraulic retention times (HRTs)) and are quick to initiate biological processes [18]. Ultrafiltration (UF) membranes have the capacity to retain SSs and most colloidal matter. Despite these advantages, membrane fouling remains a major operational problem in MBR systems, hindering their widespread implementation [18]. Various measures, such as cyclic permeate backwash, coarse bubble aeration, adjustment of operating conditions and chemical membrane cleaning, are commonly employed to mitigate fouling problems [19]. Membrane fouling in MBRs is mainly attributed to organic macromolecules, including soluble microbial products (SMPs), extracellular polymeric substances (EPSs) and potentially other compounds released during cell lysis. Among the different strategies adopted, adsorption stands out for its distinct advantages, taking advantage of synthetic and natural materials to effectively remove pollutants through chemisorption and physisorption mechanisms [20,21]. 

The incorporation of clay additives to mitigate fouling, although rarely used in large-scale MBRs, presents itself as a potentially advantageous alternative. The charge of clay minerals, governed by their ion exchange capacity, significantly influences their adsorption characteristics [22]. Clay conditioners modify the properties of the sludge and improve its filterability. Natural clay minerals typically include montmorillonite, hectorite, bentonite, sepiolite, laponite, saponite, rectorite, vermiculite, zeolite, kaolinite and chlorite [23,24]. These minerals are mainly in the form of hydrated aluminum phyllosilicates with the inclusion of iron, magnesium, alkali metals and various cations [25]. Bentonite, an economically viable material widely used in various branches of materials science, has received particular attention for its application in the treatment of wastewater containing hazardous substances [26,27]. Morocco has large reserves of bentonite in several provinces. The use of bentonite can alter the structure of flocs, reducing adhesion to the membrane. Consequently, bentonite clay serves as an excellent support, favoring an environment conducive to the growth of microorganisms capable of degrading organic matter and enhancing the denitrification process within the integrated MBR system. 

Existing studies in the technical literature generally highlight the challenge of complete nitrogen removal. The aim of this study is to examine the synergistic use of cost-effective bentonite, known for its high ion exchange capacity, in conjunction with ceramic UF membranes on a pilot scale. The aim is to improve the removal efficiency of organic and nitrogen pollutants, as well as heavy metals. Systematic evaluation of the impact of bentonite under controlled experimental conditions has made it possible to identify the most effective practices for achieving targeted removal efficiency of nutrients and metals, as well as mitigating fouling. Cost considerations are crucial, as expensive adsorbents may not be viable for wastewater treatment. There is little research on the evaluation of economic benefits or costs of wastewater treatment under practically similar conditions. In this paper, we present a new approach for evaluating the costs and benefits of the AeCMBR pilot operation in Morocco, adapted to local conditions.

## 2. Pilot-Scale AeCMBR and Operation 

**Figure 1:**
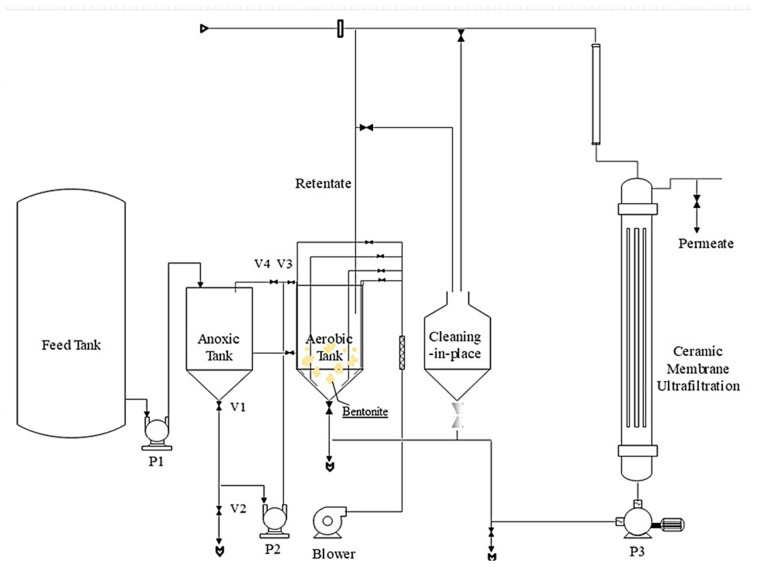
Schematic illustration of the AeCMBR pilot configuration.

A schematic illustration of the pilot-scale AeCMBR used is shown in Figure 1, which describes the integral components of the experimental setup. The integrated pilot system consists of a 200 L high-density polyethylene (PEDH) feed tank serving as a storage and feed source and an aerobic ceramic membrane bioreactor (AeCMBR) pilot unit adopting a pre-denitrification approach, which incorporates a 20 L anoxic reactor for denitrification and a 40 L oxidation reactor for nitrification. A feed pump ensures wastewater transfer from the storage tank to the reactors, while a peristaltic pump manages the effluent transfer from the anoxic tank to the aerobic reactor. The aeration tank, crucial for microbial metabolism, contains acclimatized sludge and comprises four independently controlled diffusers. The introduction of air is precisely regulated by shut-off valves, managed by a control valve. The experiment lasted over 50 days, divided into distinct stages. 

The initial phase, lasting 10 days, entailed the use of raw wastewater from a local pharmaceutical PhWW company as the feed water. Subsequent phases (I, II, III, IV) involved the addition of varying doses of bentonite into the aerobic reactor (1.5, 3, 5, 10 g/L, respectively). This dosage variation aimed to assess the related impact on improving the performance and robustness of the pilot system in terms of reducing the pollutants and heavy metals targeted, as well as reducing fouling. Throughout the investigation, the HRT remained fixed at 24 h, with no sludge withdrawal at any stage, implying a theoretically infinite SRT. Maintaining a 24 h HRT leverages bentonite’s adsorptive capabilities, enhancing biological degradation, chemical oxidation, and the adsorption processes essential for treating highly loaded wastewater. Additionally, an infinite sludge retention time (SRT) supports microbial colony development, providing flexibility during organic load fluctuations. This approach, combined with optimal COD and MLSS levels, balances microbial activity and biomass concentration, preventing excessive biomass growth and equipment clogging. 

The operating temperature within the pilot was maintained at 21.0 °C, while the dissolved oxygen concentration in the bioreactor was maintained at 3 ± 1 mg/L, corresponding to an oxygenation rate of between 1000 and 1500 NL/h. Daily monitoring of the pH of the mixed liquor revealed a fluctuation, with levels ranging from 5.2 ± 0.2 to 8.7 ± 0.3. 

The external ceramic UF membrane (Membralox^®^) (DeltaLab, Carcassonne, France), mainly made from aluminum oxide, was characterized by a tubular P10 module type, featuring a 0.45 m^2^ filtration area, 15 kD cut-off, 1178 cm length, and a 6 mm channel diameter and employing a tangential filtration approach. Permeate collection is ensured by a peristaltic pump, while retentate is recycled to the nitrification reactor through the integration of a concentric tube heat exchanger and a back pressure valve. Filtration procedures adhere to a well-defined protocol, consisting of a 42 min filtration period followed by a 3 min relaxation interval.

Transmembrane pressure (TMP) values are directly accessible using the pilot’s electrical cabinet, intricately linked to a SCADA system on the computer, allowing for the systematic recording of TMP values at specified time intervals, set at every 5 min for this study. Fouling is controlled by continuous monitoring of the TMP, with the filtration resistance calculated based on the series resistance model. Initiating a chemical cleaning process becomes imperative when the TMP threshold of 0.3–2.5 bar is reached, involving the utilization of NaOH (pH = 11) and H_2_SO_4_ (pH = 3) solutions to restore the membrane’s initial permeability.

### 2.1. Wastewater Sampling/Feeding 

PhWW samples were obtained from the wastewater treatment plant (WWTP) associated with a local pharmaceutical production factory. Raw PhWW and treated water, collected in opaque plastic drums and small bottles, respectively, were stored in a laboratory cold room at 4 °C. The treated water exiting the WWTP was collected for subsequent comparative analysis post-investigation. In addition, the sludge from the acclimatized secondary tank was sampled using a 40-litre PEDH drum. During the transportation of these samples from the plant to the laboratory, a syringe equipped with a tight fitting was intermittently employed as a manual air source. This systematic use of a syringe served to promote microbial activity and oxygenate the sample, ensuring its integrity during transit to the laboratory. 

The composition of the influent continuously feeding the bioreactors is shown in Table 1. Throughout the operational phases, a volume of 15 L of raw PhWW was fed into the reactors. The reactors were graduated, allowing precise control of the feed rate. During the subsequent solid–liquid separation stage, a continuous permeate flow of 7.5 L/h was extracted. Simultaneously, a constant flow of 30 L/h was pumped from the aerobic tank to the membrane. Moreover, a flow of recycled retentate, equal to 22.5 L/h during the solid–liquid separation phase, was constantly recycled from the membrane to the aerobic tank.

### 2.2. Preparation and Bentonite Characterization

The natural bentonite clay samples used in this study were obtained from the Larache area in the north-western region of Morocco. The preparation steps applied to the raw clay sample involved a series of operations. The clay underwent a 24 h drying period in an oven at a temperature of T = 65 °C. The material was then carefully crushed using a ceramic mortar to avoid any contamination in terms of chemical composition. The fine particles obtained were subjected to a sorting procedure with a mesh size of 63 μm. This first sorting phase not only considerably reduced large particles, but also cleared them of associated impurities such as quartz and carbonate. 

Afterwards, the removal of crystalline phases and the replacement of exchangeable cations with sodium ions was achieved by treatment with 2M NaCl, repeated five times, followed by washing and centrifugation and then sieving to obtain a powder (<2 μm particle size). The elemental composition of bentonite is summarized in Table 2. The elemental chemical analysis presented in Table 2 reveals that the raw clay is mainly composed of silica and alumina, constituting 48.7% and 21.98%, respectively. The SiO_2_/Al_2_O_3_ ratio of 2.21 indicates a significant amount of free silica in the composition. However, the clay also contains other oxides, including Fe_2_O_3_, MgO, K_2_O, and Na_2_O, collectively accounting for 6.37% of the composition. Moreover, the proportion of these oxides relative to other elements suggests the further existence of exchangeable cations, such as Mg^2+^, K^+^ or Na^+^, in the clay matrix. In addition, the low content of CaO (0.37%) in the elemental analysis indicates the limited presence of calcium carbonate in the clay.

Spectral analysis reveals a significant reduction in the intensity of absorption bands at 3422 cm^−1^ and 1639 cm^−1^, associated with the deformation vibrations of hydroxyl (OH) groups in the octahedral layer, in the purified 2 µm Na fraction. Despite this reduction, the presence of traces of dioctahedral smectites is indicated by absorption bands at 3622 cm^−1^ and 910 cm^−1^, corresponding to the elongation and deformation vibration bands of hydroxyls [Al-Al-OH] in the octahedral layer. X-ray diffraction (XRD) analysis of the raw clay and its fine fraction (<2 µm-Na) showed characteristic montmorillonite peaks at 2θ angles of 6.01°, 19.8°, 35.2° and 62.02°. The peaks show variations in intensity, suggesting a low crystallinity and reduced crystalline phases in the phyllosilicates of the raw clay. Peaks at 3.22 Å (27.68° in 2θ) and 3 Å (29.68° in 2θ) indicate the presence of quartz. The XRD results confirm that the predominant mineral in the clay and its fine fraction is a dioctahedral smectite (the results of the FTIR spectral characteristics and the crystalline structure of the raw and purified bentonite phases can be found in the Supplementary Figures S1 and S2).

At each stage of the operation, the chosen dose of bentonite for the treatment of PhWW was manually introduced into the aerobic tank, where aeration diffusers were also used to effectively disperse and mix the bentonite powder in the reactor.

### 2.3. Analytical Methods

The key parameters of the influent, mixed liquor and permeate samples were periodically assessed using standard water and wastewater analysis methods [28]. Measurements of chemical oxygen demand (COD) were carried out with a Hach DR2800 spectrophotometer, using the spectrophotometric method based on the determination of excess potassium dichromate at 600 nm. Total Kjeldahl nitrogen (TKN) was determined using Kjeldahl methods [28], and the nitrate content was measured using an electrode (Sension MM 340). Dissolved oxygen (DO), pH and temperature were measured using a WTW-Multi 340i multimeter (Germany). A JENWAY pH meter was also used to measure pH, while a multiparameter conductivity meter (inoLab) was used to determine electric conductivity (EC). Phosphate ion concentrations were measured using a colorimetric method based on complex formation with ammonium molybdate and potassium antimony tartrate in acidic solution [29].

In this study, the underlying hypothesis was that irreversible fouling could be linked entirely to soluble microbial products (SMPs). Filtration techniques were systematically employed to measure SMP concentrations in AeCMBR supernatants, involving filtration through cellulose acetate syringe filters with a pore size of 0.45 µm. Inductively Coupled Plasma Optical Emission Spectroscopy analysis (ICP-OES, Perkin Elmer Optima 8000, Waltham, MA, USA) was used to quantify the concentrations of Cu, Ni, Pb and Zn. The chemical composition of bentonite samples was determined using an X-ray Fluorescence Spectrometer (XRF, panalytical Axios FAST simultaneous WDXRF). The Fourier transform infrared (FTIR) spectrum of bentonite was obtained using a JASCO Asia Portal FT/IR-6600 spectrophotometer (Jeddah, Saudi Arabia). X-ray diffraction (XRD) analysis to detect the crystalline phases in the raw and purified bentonite samples was carried out using an XRD diffractometer (X’Pert Pro MPD, PANalytical, Almelo, The Netherlands). The methylene blue (MB) technique was used to measure the specific surface area (SSA) of bentonite, which highlights the amount of MB adsorbed on the surface area of bentonite [30].

### 2.4. Membrane Fouling Control

The series resistance model was used for the study of specific fouling mechanisms, applying the same method in our previous study following a procedure modified by Mannina et al. [31,32]. Membrane resistance was determined using the Darcy equation:

(1) $$R=\frac{TMP}{\mu J}$$

where J is the flux in m^3^/(m^2^·s), TMP is the transmembrane pressure in Pa, μ is the permeate viscosity in (Pa·s), and R is the membrane filtration resistance in 1/m.

For the AeCMBR pilot plant, a cycle of 42 min of filtration followed by 3 min of relaxation was used. During relaxation, it was assumed that all reversible fouling was removed, creating a strong shear effect during backwashing. The steady-state TMP data within the 1 to 2 h operational duration under each condition were used to compute the reversible fouling rate, rf:

(2) rf=ΔTMP∕ΔT

The total filtration resistance was determined by:

(3) RT=Rm+Rr+Rirr

where Rt is the total resistance, Rm is the intrinsic membrane resistance, Rr is the reversible fouling resistance, and Rirr is the irreversible fouling resistance.

It was assumed that all reversible fouling was removed during relaxation, so the TMP immediately after relaxation (TMP_0_) was due to Rm and Rirr:

(4) $$R_{m}+R_{irr}=\frac{TMP_{0}}{\mu J}$$

The TMP changes just before and after relaxation were attributed to the reversible fouling resistance, Rr:

(5) $$R_{r}=\frac{TMP_{1}-TMP_{0}}{µJ}$$

where TPM_1_ is the TMP immediately before relaxation and TMP_0_ is immediately after. This methodology allowed for the calculation of membrane resistance, Rm, and reversible fouling resistance, Rr.

## 3. Pilot-Scale AeCMBR–Bentonite Performance

### 3.1. Effect on COD Removal 

**Figure 2:**
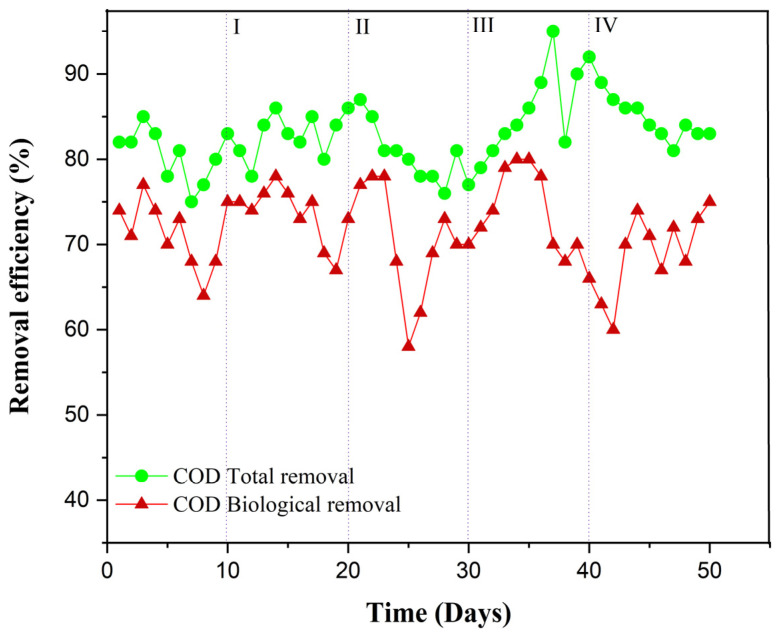
COD concentration in AeCMBR–bentonite supernatant and effluent.

The AeCMBR–bentonite has shown a stable and effective performance in terms of fundamental water quality parameters and operational aspects. COD, a crucial indicator of organic pollution in wastewater, is used as a benchmark to assess the effectiveness of the treatment processes applied. Figure 2 shows the removal efficiency of COD. In the initial start-up phase, without the addition of bentonite, the total COD removal averaged 80.40%, with a maximum biological reduction of 77% during the first 5 days. However, in phases I and II, characterized by the addition of bentonite concentrations of 1.5 g and 3 g, respectively, the overall COD removal efficiency increased to 82.9% and 81.3%, respectively, with the lowest biological abatement of around 58% observed throughout the experiment. This outcome may be attributed to the colloidal structure of bentonite and its electrokinetic properties. The significant flocculation observed during these phases considerably reduced the concentration of organic colloids in the mixed liquor.

In phase III (5 g), there was a notable increase in biological COD removal efficiency to an average of 73%, suggesting an enhancement in biological degradation facilitated by bentonite. The addition of 5 g of bentonite corresponded to an average removal of 84.27%, approximately 3% higher than the previous phase, indicating the high absorption capacity of the well-domesticated sludge during this stage. However, in the final phase marked by the addition of 10 g of bentonite, biological removal dropped to 60% on day 43. This decline can be attributed to the fact that COD has no anionic or cationic charges for adsorption onto bentonite, and there is no potential for exchange with its cations. In summary, stable COD removal was observed with the addition of bentonite doses, indicating a low sorption of organic matter. The utilization of bentonite (1.5, 3, 5, 10 g/L) did not yield a significant reduction in the COD compared to the start-up phase without bentonite. The maximum reduction was achieved with the use of 5 g.

This leads to the conclusion that bentonite has no substantial impact on COD removal, as organic carbons, such as COD, have no anionic or cationic charges to remove. This aligns with findings from other researchers who have demonstrated that minerals like zeolite, montmorillonites, and perlite do not possess the potential to enhance COD removal [33,34].

### 3.2. Effect of Bentonite on Nitrogen Removal

#### 3.2.1. Ammonia Nitrogen Levels

**Figure 3:**
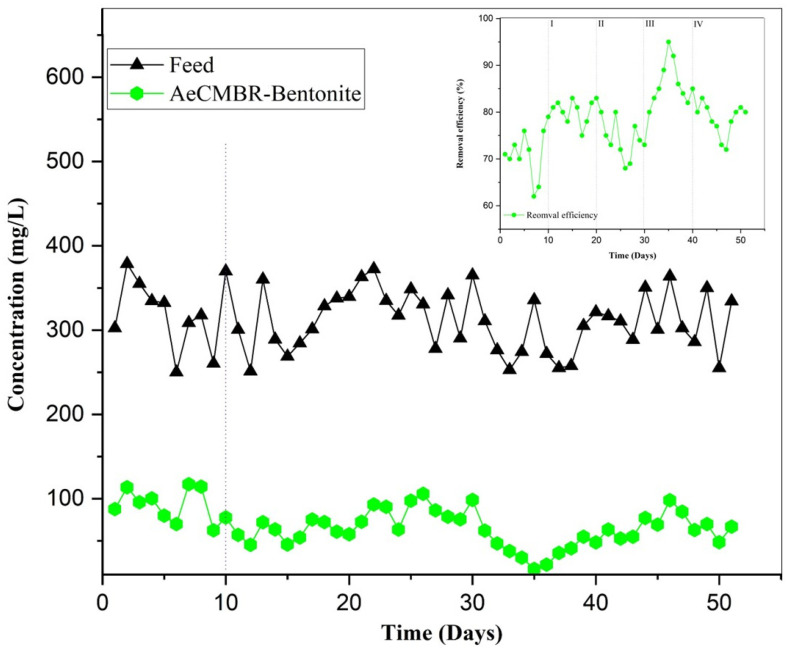
NH4+ removal performance of the AeCMBR–bentonite system.

The PhWW studied had a high N-NH4 content, more than 312 mg/L. Bentonite particles, characterized by a negatively charged surface and a high cation adsorption capacity, were used because of their potential to provide an environment conducive to the supply of nutrients to nitrifiers. Figure 3 illustrates the efficiency of ammoniacal nitrogen removal and concentration reduction throughout the experimental period. The average influent concentration during the initial start-up phase (first 10 days) was 321.029 mg/L, decreasing thereafter to an average effluent concentration of 91.92 mg/L. This results in a removal efficiency of 71%. N-NH_4_^+^ was eliminated during this stage only via a biological pathway, given that the sludge was well acclimatized.

During phase I, characterized by the addition of 1500 mg of bentonite, the removal efficiency increased to 80.3%, with an average reduction in concentration from 306.208 to 60.39 mg/L. Nitrifiers prefer attached growth; thus, the biofilm formed on the particles was mainly made up of this type of bacteria. This favors the nitrification reaction and adsorption. During phase II, the average removal efficiency achieved was 74.2%. Despite the increase in bentonite to 3000 mg/L, the removal efficiency dropped significantly by around 6% compared with the previous phase, and no further reduction was observed. This suggests slow cation exchange with the bentonite cations, while biological removal predominated.

In Phase III (5 g/L bentonite), the concentration decreased from 286.19 mg/L to 39.59 mg/L, reaching an average removal efficiency of 86.16%. The plasticity of the bentonite, along with the dosage applied and aeration conditions during this phase, significantly enhanced the retention of NH4+ cations in the bentonite exchange sites, thereby promoting effective adsorption. The average removal efficiency in the final phase was 78.36%. The maximum reduction observed reduced the influent concentration from 321.46 mg/L to 48.219 mg/L in the effluent, while the minimum reduction on day 46 reduced the ammonium concentration from 363.91 mg/L to 98.25 mg/L. Doubling the concentration of bentonite added in this phase did not result in any significant improvement in removal efficiency. This lack of improvement was attributed to the need for the bacteria to adapt over a longer period, given the progressive saturation of the surface of the bentonite particles with different ions and cations during the previous phases.

The average NH_4_^+^ removal rate throughout the survey was 78%. As a result, the nitrification performance in the AeCMBR system was found to be higher than values reported by other researchers [35,36]. In general, the addition of bentonite to activated sludge during operation facilitated the growth and activity of nitrifying bacteria, thereby improving the removal of potential inhibitors that could otherwise have adverse effects on nitrification.

#### 3.2.2. Nitrate Variation

**Figure 4:**
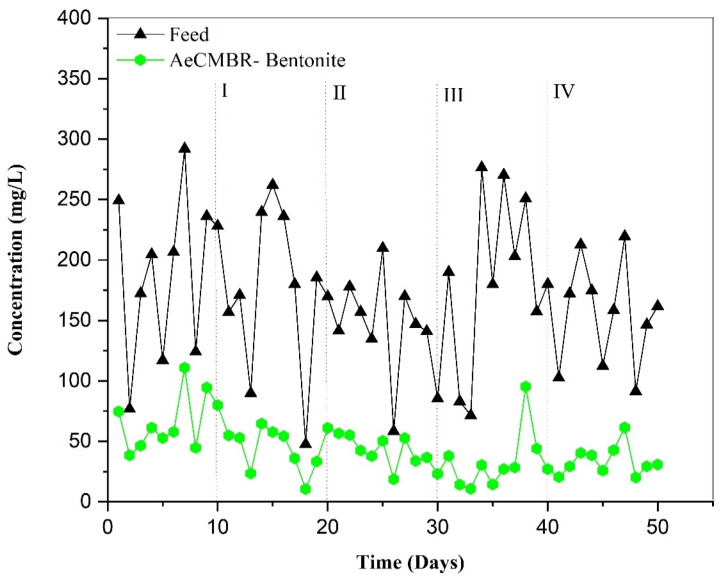
The variation in NO_3_ removal during operation.

Sustaining the activity of the bacterial community, especially denitrifying species, for a complete reduction of nitrogen depends on a sufficient source of carbon. Maintaining a COD/NOx-N ratio within the range of 2.5 to 6.0 is essential for complete reduction of NOx-N to elemental nitrogen [37]. It is noteworthy that pharmaceutical production at the source plant involves the use of nitro compounds in its preparations. In this study, the use of a high-carbon feedwater, with a COD greater than 3780 mg/L and a C/N ratio of 5.02, enabled almost complete removal of NOx-N via a biological pathway. Figure 4 illustrates the fluctuation in nitrate in the AeCMBR–bentonite system, showing the concentration of nitrate in the effluent relative to the feed water during each operational phase. Over the first 10 days, influent nitrate levels in the system ranged from 76.88 to 249.1 mg/L, resulting in an average removal efficiency of 65%. There was a noticeable accumulation of nitrate in the effluent, reaching a maximum value of 110.96 on day 7. This could be attributed to a disruption in the conversion of ammonia to nitrate, given two 2 h interruptions (power cuts) to the aeration system during the first 5 days.

During phases I and II, the average nitrate concentrations were 173.85 mg/L and 142.33 mg/L, respectively. These concentrations were reduced in the effluent to 44.89 mg/L and 40.72 mg/L, respectively, suggesting that the added bentonite could establish an anoxic environment favorable for denitrifying bacteria, given its porous structure [38]. In phase III, given the cation exchange capacity of the bentonite with NH_4_^+^, nitrification increased by 38% compared with the initial 10 days, leading to elevated nitrate production, reaching a concentration of 95.33 on day 38. Figure 4 shows that after the addition of bentonite, from day 11, there was a gradual decrease in nitrate levels until day 33, followed by an increase in nitrate production until the end of the experiment. This fluctuation indicates a period of saturation during which no adsorption occurred, and the microorganisms were only able to eliminate part of the excess nitrate, leaving the rest suspended.

This study highlights the importance of adding bentonite to facilitate the formation of biofilms, a critical factor in creating an anaerobic environment suitable for denitrification. It should be noted that the depth of oxygen permeation through a biofilm typically ranges from 100 to 150 μm [39], requiring a thicker layer to establish optimal anaerobic conditions. Unfortunately, the depth of the biofilm on the bentonite used in this experiment was not measured, making it difficult to specify whether it is within the optimal range for anaerobic conditions, given that the experiment was conducted with a short duration. Therefore, future studies could further explore the effect of biofilm formation on particles under prolonged conditions. 

### 3.3. Bentonite Effect on Heavy Metal Removal

**Figure 5:**
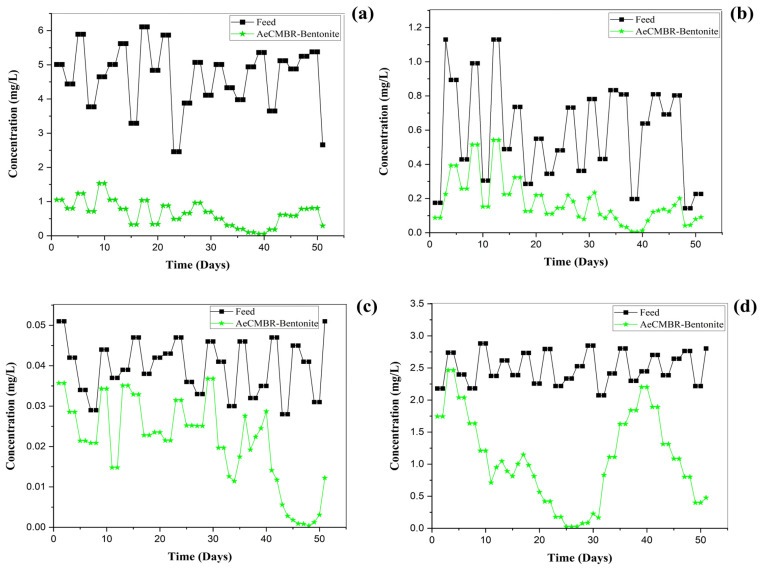
Adsorption behaviors of (**a**) Cu, (**b**) Ni, (**c**) Pb, (**d**) Zn in the AeCMBR–bentonite system.

Figure 5 illustrates the variation in heavy metal concentrations (mg/L) adsorbed by bentonite based on the initial concentrations (Cu, Ni, Pb, and Zn) in the feedwater and the corresponding removal efficiencies in the AeCMBR–bentonite system. Analyses of these heavy metals were conducted bi-daily. The results show a significant reduction in Cu concentrations in the effluent, with the average influent concentration of 4.65 mg/L reduced to an effluent concentration of 0.454 mg/L. This results in a removal efficiency of over 90%, indicating effective diffusion of cations onto the specific surface of the examined bentonite. The optimum dose of bentonite, resulting in superior adsorption results for Cu metals, was 5000 mg/L, achieving removal efficiencies in excess of 94% during phase III. The impact of bentonite on the adsorption capacity for Ni metals closely resembles that observed for Cu. As depicted in Figure 5b, the results indicate an increase in the removal efficiency of Ni, corresponding to an increased mass of added bentonite. The adsorption reached its maximum on days (34–37), i.e., the pH during this period was greater than 7, and such an adsorption capacity was due to the complexation of the outer sphere and the inner sphere formed by the hydroxides of Ni metals, which may have bonded to the silanol and aluminol sites of the bentonite, a finding which has been demonstrated by other previous studies [40,41].

Figure 5c shows that the Pb concentration in the influent was partially stable and that a gradual decrease in Pb concentration was well established, reaching a maximum during the final phase. During the first stages, various forms were generated as a function of pH (5–7) and the concentration of metal ions (Pb). For instance, pb^2+^ metal ions predominate at a low pH, and Pb hydroxide begins to form at around a pH of 6–7; precipitation was only dominant at pH > 6. Throughout the experiment, the average Pb removal efficiency was around 46%. The limited adsorption capacity under acidic conditions is attributed to competition between metal ions and H_3_O^+^ for adsorption sites. In addition, the formation of positively charged aquatic complexes on the bentonite surface leads to electrostatic repulsion of metal ions [42,43]. In the final phase (days 40–50), the adsorption capacity increased with pH (6–8), mainly through ion exchange and complexation of the outer sphere and inner surface. These conclusions contradict the results of other studies on Pb adsorption on montmorillonite and those published by Tongjiang et al. (2006) [44]. 

The adsorption efficiency of heavy metals is strongly influenced by the ligand content of the medium. In addition, bentonite shows greater selectivity for Pb than for Zn, and Cu was less effective at displacing Pb from adsorption sites than Pb was at displacing Cu [45].

In Figure 5d, the influent Zn concentration remained practically stable throughout the operation, measuring 2.49 mg/L with a standard deviation of 0.21. The startup phase and the subsequent two phases (1.5, 3 g/L of bentonites, respectively) show a gradual decrease in the concentration of Zn in the effluent. During phases III and IV, with a pH of 7.8 ± 0.9, Zn adsorption was impacted due to its low solubility in a mildly alkaline environment. This could be attributed to the formation of soluble hydroxyl complexes under these conditions, which leads to competition for adsorption sites and consequently reduces adsorption efficiency [46].

### 3.4. Bentonite Concentration’s Effect on Membrane Resistance

Monitoring membrane fouling involved tracking transmembrane pressure (TMP) values, with resistance values determined by Dracy’s law:

(6) $$R=\frac{TMP}{\mu J}$$

where J signifies the flux in m^3^/(m^2^·s), TMP represents the transmembrane pressure in Pa, µ stands for the permeate viscosity in (Pa·s), and R denotes the membrane filtration resistance as 1/m.

**Figure 6:**
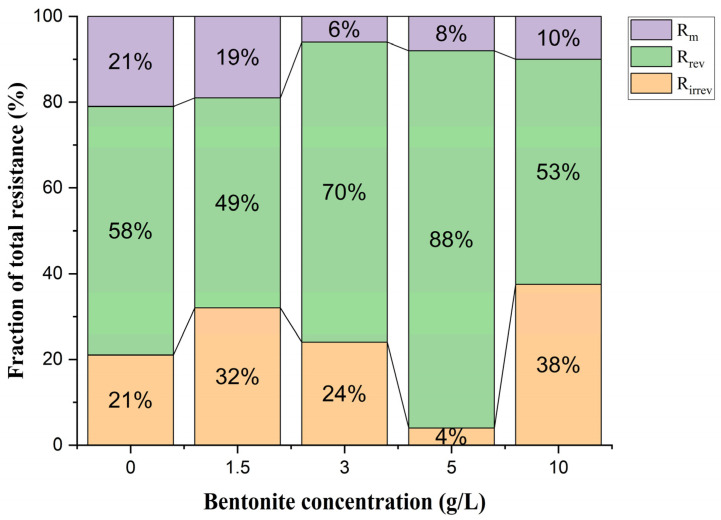
Total membrane resistance as a function of bentonite concentration.

The overall filtration resistance was evaluated using the series model R_T_ = R_m_ + R_r_ + R_irrev_ [31]. Figure 6 illustrates the distribution of resistance fractions (Rm, membrane resistance; R_rev_, reversible resistance; and R_irrev_, irreversible resistance) measured for different bentonite feed rates throughout the experiment. During the investigation, increasing the dosage of bentonite had a positive impact on membrane filtration stability.

In the initial phase, the total resistance was 6.2 × 10^12^ m^−1^, with 58% attributed to the deposition of the cake layer, subsequently removed by backwashing. At the same time, an increase in resistance associated with pore clogging was observed with increasing bentonite concentrations. In the 1.5 g/L bentonite addition phase, the total resistance reached 6.7 × 10^12^ m^−1^, with the irreversible fraction measuring 2.14 × 10^12^ m^−1^. The trend towards irreversible fouling may have been influenced by the SMP concentration exceeding 965 mg/L on days 1–20, which resulted in a reduction in membrane flux to 6.5 LMH (day 18).

One possible interpretation is that the interaction between the mixed liquor emulsion and the bentonite governs the adsorption of the cake layer onto the membrane surface, resulting in a decrease in membrane flux. Following this observation, and in the next relaxation step, NaOH solution (pH = 11) was introduced from the clean-in-place tank to the membrane for 5 min, followed by a rinse with tap water for 2 min, then H_2_SO_4_ solution (pH = 3) for 5 min and a final rinse with tap water for 3 min for cleaning purposes.

In phase II (3 g/L bentonite), the total resistance dropped by around 18% (RT = 5.5 × 10^12^ m^−1^), leading to a decrease in the irreversible resistance (Rirrev) to 1.32 × 10^12^ m^−1^, characterized by a lower resistance compared with the reversible resistance and the gradual deposition of the cake layer. These results could be correlated with the impact of the bentonite on the properties of the SMP, which showed bloating, a high hydrophobicity, and agglomeration in the settled sludge. Bentonite improved the overall resistance by reducing the floc size and attenuating the adhesion and viscosity of the organic matter. Nevertheless, in phase III, fouling mainly attributed to the dominated cake layer (88%). As indicated by Meng et al. (2008) [47], smaller particles and colloids have a greater propensity to deposit on the membrane surface. In addition, previous investigations have shown that particles smaller than 50 µm lead to an increase in the specific resistance of the cake layer [48,49]. Temperature plays a crucial role in the dissolution of these particles. Cong et al. found that the total membrane resistance was 0.25 × 10^12^ m^−1^, with reversible resistance (Rrev) accounting for around 83% at 10 °C and 89% at 20 °C of the total resistance (Rt) [50]. Practical operating time at high adsorbent concentrations is largely dependent on the ratio of irreversible resistance (Rirr) to Rt, which increases with decreasing temperature, consistent with previous studies [51,52,53]. 

The intrinsic resistance of the ceramic membrane is 1.7 × 10^12^ m^−1^. The observed values for membrane resistance (Rm) throughout all operational phases distinctly indicate a stable reduction in permeability achieved with the addition of bentonite. The total resistance during start-up and bentonite addition phases was 6.2 × 10^12^, 6.7 × 10^12^, 5.5 × 10^12^, 5.2 × 10^12^, and 5.9 × 10^12^ m^−1^, respectively. This observation aligns with the findings of Panpanit et al., who investigated the treatment of oil/water emulsions from car wash wastewater using ultrafiltration. Their study demonstrated that the total membrane resistance (Rt) decreases with bentonite concentrations in the oil emulsion ranging from 0 to 300 mg/L. However, at concentrations above 600 mg/L, the membrane resistance Rt significantly increases [54].

Increasing the bentonite concentration from 5 g/L to 10 g/L increased the resistance from 5.2 × 10^12^ to 5.9 × 10^12^ m^−1^, probably due to deflocculation caused by the bentonite elements acting as a coagulant. Malamis et al. examined the impact of three natural minerals (bentonite, zeolite and perlite) and found that high concentrations of coagulants resulted in a positive charge of the biomass, which favored partial deflocculation and increased colloidal matter [33]. Similarly, Song et al. observed that increasing Al (III) from 23.7 to 39.5 mg/L increased the specific resistance of the cake layer from 6.68 × 10^12^ to 2.23 × 10^19^ m^−1^ [55].

At 5 g/L of bentonite (Phase III), the SMP concentration dropped to 295.45 mg/L. The experiment identified the optimal bentonite concentration for fouling mitigation, proving more effective at 5 g/L than at 10 g/L bentonite addition. This is likely due to the significant increase in the overall solid concentration in the sludge at the 10 g/L concentration. The total membrane resistance was primarily influenced by the formation of a layer of deposited bentonite particles rather than the adsorption of organic foulant particles on the membrane surface. Despite this, the addition of bentonite resulted in a significant reduction in membrane fouling at different concentrations. Colloidal matter and soluble microbial products are recognized as the main contributors to membrane fouling in MBR systems [56,57]. The results indicate that the bentonite examined has a considerable surface/volume ratio, which allows for effective adsorption of colloidal matter and SMPs from sludge onto its surface.

## 4. Cost Implication and Sustainable Considerations

### 4.1. Economic Analysis

A cost analysis of adding bentonite, a natural and inexpensive material, to the aerobic membrane bioreactor (AeCMBR) was conducted using an effective and prospective method. This approach evaluates cost-effectiveness and financial feasibility based on the formulas below by combining methods adapted from previous studies [58,59,60]. The analysis overlooks the Net Present Value (NPV) approach and the potential for bentonite regeneration. The economic assessment was performed to define the implication on cost based on pilot-scale experiments and extrapolation to actual industrial production. This research provides comprehensive data on operating costs during the experimental period and implication costs for purifying and adding bentonite, aiming to enhance the processing performance of the AeCMBR.

Table 3 summarizes the details of the components used in the cost analysis. To perform a detailed economic analysis of adding bentonite clay to the AeCMBR, we examine the various cost elements involved over the operating period (O.P). The total cost of bentonite clay (TCB) is calculated by multiplying the unit cost of bentonite (CB) by the total quantity (QB) used during the O.P. Additionally, the total operational costs (TOCs) include the energy cost for mixing and handling bentonite (CE) and the labor cost for preparation and addition (CL). Maintenance costs (NMCs) cover expenses associated with cleaning (CM), including chemical reagents (NaOH and H_2_SO_4_), and are offset by savings from reduced fouling (SF), which decreases the frequency of cleaning and extends membrane life. Improved performance savings are derived from an enhanced water quality (TPIS), which results in benefits such as reduced penalties and an improved effluent quality. Data for this analysis were extrapolated in coordination with the person in charge of the wastewater treatment plant (WWTP) at the pharmaceutical factory being investigated.

(7) Total Bentonite Cost (TCB)=CB×QB

(8) $$Total\;Operation\;Cost\left(TOC\right)=CE+CL+CM+TCB$$

(9) $$Net\;Maintenance\;Cost\left(NMC\right)=TOC-SF$$

(10) Total Performance Improvement Savings (TPIS)=SF+SWQ

(11) $$Net\;Cost\;savings\left(NCS\right)=TPIS-TOC$$

(12) $$ROI=\frac{NCS}{TOC}\times 100$$

The total bentonite cost (TCB) is USD 0.24 per operating period (O.P.), calculated by multiplying the unit cost of bentonite (USD 1.27/kg) by the quantity used (0.195 kg/O.P). The total operational cost (TOC) is USD 55.24 per O.P., which includes energy costs (USD 20), labor costs (USD 10), membrane cleaning and replacement costs (USD 25), and the TCB (USD 0.24). The net maintenance cost (NMC) is USD 20.19 per O.P., derived from subtracting savings from reduced fouling (USD 35.05) from the TOC.

The total performance improvement saving (TPIS) is USD 75.05 per O.P., combining savings from reduced fouling (USD 35.05) and improved water quality savings (USD 40). The net cost/saving (NCS) is USD 19.80 per O.P., calculated by subtracting the TOC from the TPIS. The payback period (PP) is not applicable since there is no initial investment specified.

For the economic analysis, we started by calculating the initial investment, which includes the total cost of the bentonite and any necessary modifications to the system. The operational cost is then calculated as the sum of the total operational cost and the net maintenance cost. Savings are derived from total performance improvement savings. The net cost or saving is the difference between savings and operational costs. The payback period, which is the time required to recoup the initial investment from the net savings, is calculated by dividing the initial investment by the net cost or savings over the operating period. Finally, the return on investment is calculated to measure the profitability of the investment, expressed as a percentage.

In summary, the economic analysis of adding bentonite to the aerobic membrane bioreactor shows a total operational cost (TOC) of USD 55.24 per O.P and total performance improvement savings (TPISs) of USD 75.05 per O.P. The net cost/saving (NCS) is USD 19.80 per year, indicating a net saving. The return on investment (ROI) is 35.83%, suggesting a favorable return due to the performance improvements and cost savings from the bentonite addition process. 

### 4.2. Sustainable Considerations

In our study, bentonite plays a crucial role in enhancing the wastewater treatment process and mitigating fouling. It functions as an adsorbent, effectively removing contaminants from the wastewater and adsorbing precursor particles such as soluble microbial products that can cause fouling, ensuring that the system functions properly. Bentonite’s dual functionality as an adsorbent and ion exchanger adds versatility, enabling it to trap and retain ions and contaminant molecules through its large specific surface area and negative charge, while also facilitating the removal of specific metal ions (Cu, Pb, Zn, Ni) through ion exchange. This multifaceted approach not only improves the treatment efficiency but also offers economic benefits.

To study the long-term operational feasibility of using bentonite in AeCMBR systems, several aspects need to be considered: Regeneration potential. The feasibility and effectiveness of regenerating spent bentonite should be investigated to prolong its usage lifespan. This includes studying regeneration methods, such as thermal or chemical treatments, and assessing their economic and environmental implications. Previous studies have demonstrated the reuse of bentonite through adsorption–desorption cycles, typically involving treatment with 4% HCl for 2 h, followed by rinsing with deionized water until a neutral pH is achieved [61,62,63,64]. Marszałek et al. found high reuse potential for bentonite-based adsorbents, with percentage adsorption capacities remaining high even after four cycles of use. For example, the capacities of Cu (II) columns ranged from 30% to 73%, and those of Pb(II) columns from 50% to 76%, indicating efficient removal of heavy metal ions from aqueous solutions [64].

When addressing the key sustainability considerations associated with the use of bentonite in AeCMBR systems, it is essential to carry out a thorough assessment of the long-term operational feasibility of bentonite. This involves assessing its environmental impact, its extraction process and its potential ecological footprint. Recent studies, such as that by Satyannarayana et al., have examined the manufacture of a tubular ceramic membrane from local bentonite and have highlighted the importance of taking environmental factors into account in membrane processes. Their findings highlight that the source of electricity has a significant impact on environmental indicators, with the move away from conventional energy sources resulting in substantial reductions in environmental impacts [65]. These observations highlight the need to adopt sustainable practices in the use of materials such as bentonite in AeCMBR systems in order to minimize environmental consequences and ensure long-term viability.

## 5. Conclusions

This study explored the use of powdered bentonite as an accessible and cost-effective natural adsorbent to enhance the removal of pollutants from PhWW using an AeCMBR process. The pilot-scale AeCMBR has operated successfully, indicating that the adsorption process has improved the efficiency of PhWW treatment. Analysis of the experimental data led to the following conclusions:

- The average COD removal throughout the experiment reached 83%. However, upon comparing results across different phases, it was observed that bentonite had no substantial impact on COD removal.
- The addition of bentonite to the AeCMBR system created a more favorable environment for improving biological degradation. In particular, the cation exchange capacity of the bentonite complemented the treatment, leading to greater NH4+ removal. Although nitrate removal remained relatively stable throughout the experiment, the formation of a biofilm layer could potentially produce greater reductions if the system was operated for longer periods under more anoxic conditions.
- Bentonite showed a considerable removal capability towards metals (Cu, Ni, Pb and Zn) contained in the studied PhWW. The average removal rates for Cu, Ni, Pb and Zn throughout the process were 86%, 68.52%, 46.90% and 56.76%, respectively. The adsorption behavior of heavy metals is closely linked to the mineralogical composition of the bentonite, its cation exchange properties and the presence of different ionic forms at different pH levels.
- The results show that the use of bentonite is a viable strategy for reducing membrane fouling, with a significant reduction observed at a concentration of 5 g/L.
- In conclusion, the economic analysis reveals a net saving of USD 19,80 over a 50-day operating period, with a favorable return on investment (ROI) of 35.83%. These results underline the cost-effectiveness and financial viability of incorporating bentonite into aerobic membrane bioreactors, through improved performance and cost savings.

In summary, this study has developed an integrated approach that effectively removes pollutants from PhWW, addressing the challenges posed by high loads of organic pollutants, nitrogen compounds and heavy metals. This solution offers a practical and effective method for industries treating high strength effluent, ensuring compliance with stringent regulatory standards [66,67,68].

## Figures and Tables

**Table 1:** Main characteristics of raw wastewater.

Parameters	Value	Standard Deviation
pH	6.95	1.75
EC (mS/cm)	13.87	1.27
Turbidity (NTU)	96.55	10.7
COD (mg/L)	3780.33	228
BOD (mg/L)	1497.61	118.26
NH_4_^+^ -N (mg/L)	312.03	37.01
NO_3_^−^ -N (mg/L)	169.69	59.86
TKN (mg/L)	536.87	84.76
PO_4_^3−^ -P (mg/L)	8.89	1.05
SO_4_^2−^ (mg/L)	108.03	17.13
TSS (mg/L)	233.82	64.34
Cu (mg/L)	4.67	0.90
Ni (mg/L)	0.59	0.29
Pb (mg/L)	0.039	0.006
Zn (mg/L)	2.49	0.24

Measurements were made almost daily and are expressed as mean ± SD (*n* = 3).

**Table 2:** Main composition and characteristics of bentonite.

Element	Concentration (%)
SiO_2_	48.70
Al_2_O_3_	21.98
CaO	0.3754
Fe_2_O_3_	0.8951
MgO	3.0908
K_2_O	0.249
Na_2_O	2.008
TiO_2_	0.0985
P_2_O_5_	0.05744
Cl	0.6399
SO_3_	0.2596
SrO	0.009995
LOI ^a^	8.870
ZrO_2_	0.047
Surface area	62 m^2^/g

^a^ Loss on ignition.

**Table 3:** Estimated cost of involvement of bentonite in the AeCMBR.

Cost Components	Cost (USD)
Unit Cost of Bentonite (CB)	USD 1.27/kg
Quantity of Bentonite Used (QB)	0.195 kg/year
Energy Cost for Mixing (CE)	USD 20/year
Labor Cost (CL)	USD 10/year
Membrane Cleaning and Replacement Cost (CM)	USD 25/year
Savings from Reduced Fouling (SF)	USD 35.05/year
Improved Water Quality Savings (SWQ)	USD 40/year

## Data Availability

The original contributions presented in the study are included in the article; further inquiries can be directed to the corresponding author.

## Supplementary Materials

The following supporting information can be downloaded at: https://www.mdpi.com/article/10.3390/membranes14100205/s1, Figure S1: FTIR spectra of raw clay samples (Bentonite) and its fine homoionized fraction; Figure S2: Illustration of X-ray diffraction of raw montmorillonite and its sodium fraction.
