# Supplementary material for: Enhanced Pollutant Removal and Antifouling in an Aerobic Ceramic Membrane Bioreactor with Bentonite for Pharmaceutical Wastewater Treatment
Source: Membranes (Basel). 2024 Sep 26;14(10):205. doi: 10.3390/membranes14100205 (PMC11509799; doi:10.3390/membranes14100205)
Supplement: Supplementary file 1 [file membranes-14-00205-s001.zip › membranes-3018964-supplementary.pdf]

Supplementary Material

Article

Enhanced Pollutant Removal and Antifouling in an Aerobic Ceramic Membrane Bioreactor with Bentonite for Pharmaceutical Wastewater Treatment

Salaheddine Elmoutez [1,*], Hafida Ayyoub [2], Mohamed Chaker Necibi [1], Azzedine Elmidaoui [1] and Mohamed Taky [2]

[1] International Water Research Institute IWRI, Mohammed VI Polytechnic University, Lot 660, Ben Guerir 43150, Morocco; chaker.necibi@um6p.ma (M.C.N.); azzeddine.elmidaoui@um6p.ma (A.E.)

[2] Laboratory of Advanced Materials and Process Engineering, Faculty of Sciences, Ibn Tofail University, Kenitra BP 1246, Morocco; hafida.ayyoub@uit.ac.ma (H.A.); mohamed.taky@uit.ac.ma (M.T.)

* Correspondence: salaheddine.elmoutez@um6p.ma

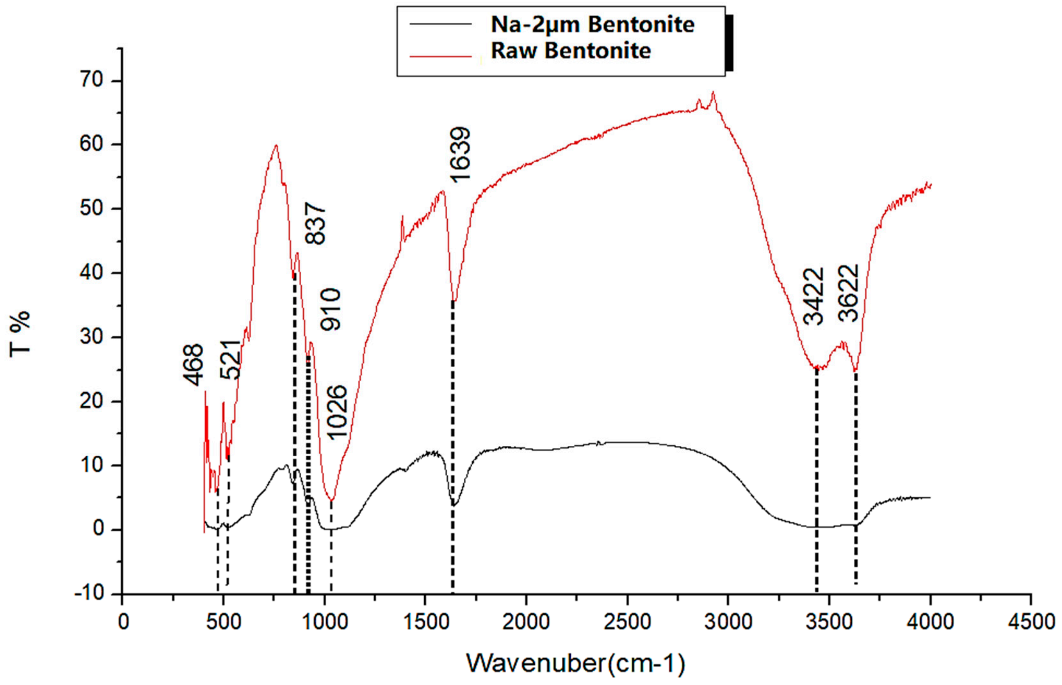

Figure S1: FTIR spectra of raw clay samples (Bentonite) and its fine homoionized fraction (2µm-Na).

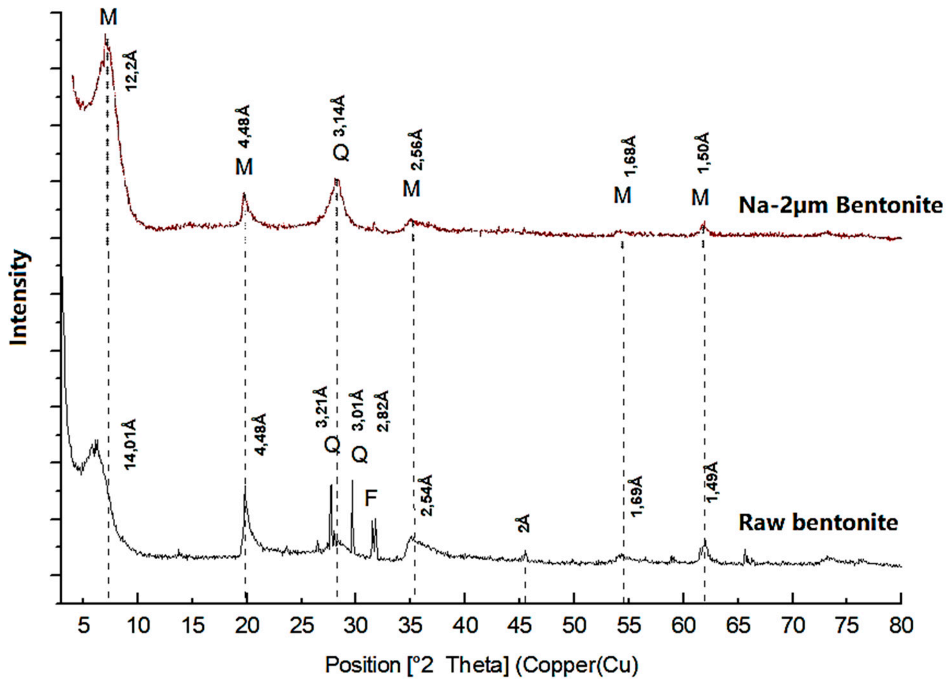

Figure S2: Illustration of X-ray diffraction of raw montmorillonite and its sodium fraction of 2µm-Na (Q = quartz, M = montmorillonite and, F = Feldspar).
